# Function modification of SR-PSOX by point mutations of basic amino acids

**DOI:** 10.1186/1476-511X-10-59

**Published:** 2011-04-15

**Authors:** Weiwei Liu, Lan Yin, Chunxia Chen, Yalei Dai

**Affiliations:** 1 Department of Immunology, Tongji University School of Medicine, 1239 Siping Road, Shanghai, 200092, China

## Abstract

**Background:**

Atherosclerosis (AS) is a common cardiovascular disease. Transformation of macrophages to form foam cells by internalizing modified low density-lipoprotein (LDL) via scavenger receptor (SR) is a key pathogenic process in the onset of AS. It has been demonstrated that SR-PSOX functions as either a scavenger receptor for uptake of atherogenic lipoproteins and bacteria or a membrane-anchored chemokine for adhesion of macrophages and T-cells to the endothelium. Therefore, SR-PSOX plays an important role in the development of AS. In this study the key basic amino acids in the chemokine domain of SR-PSOX have been identified for its functions.

**Results:**

A cell model to study the functions of SR-PSOX was successfully established. Based on the cell model, a series of mutants of human SR-PSOX were constructed by replacing the single basic amino acid residue in the non-conservative region of the chemokine domain (arginine 62, arginine 78, histidine 80, arginine 82, histidine 85, lysine 105, lysine 119, histidine 123) with alanine (designated as R62A, R78A, H80A, R82A, H85A, K105A, K119A and H123A, respectively). Functional studies showed that the mutants with H80A, H85A, and K105A significantly increased the activities of oxLDL uptake and bacterial phagocytosis compared with the wild-type SR-PSOX. In addition, we have also found that mutagenesis of either of those amino acids strongly reduced the adhesive activity of SR-PSOX by using a highly non-overlapping set of basic amino acid residues.

**Conclusion:**

Our study demonstrates that basic amino acid residues in the non-conservative region of the chemokine domain of SR-PSOX are critical for its functions. Mutation of H80, H85, and K105 is responsible for increasing SR-PSOX binding with oxLDL and bacteria. All the basic amino acids in this region are important in the cells adhesion via SR-PSOX. These findings suggest that mutagenesis of the basic amino acids in the chemokine domain of SR-PSOX may contribute to atherogenesis.

## Background

Atherosclerosis (AS) is a common cardiovascular disease that threatens human health. The pathological features of AS include infiltration of monocytes, formation of foam cells and proliferation of smooth muscle cells [1-4]. The first macroscopically recognizable lesion of atherosclerosis arises from the accumulation of macrophage-derived foam cells with cholesterylester-rich lipid droplets [5]. A high level of plasma concentration of low-density lipoprotein (LDL) is an important risk factor for atherosclerosis. When LDL molecules are trapped in an arterial wall, they are modified or oxidized and internalized by macrophage via a relatively large family of scavenger receptors (SR) [6-8] including scavenger receptor class A (SR-A), CD36 [9], CD68 [10], the lectin-like oxLDL receptor [11] and scavenger receptor for phosphatidylserine and oxidized lipoprotein (SR-PSOX) [12]. Monocytes/macrophages engulf oxLDL via the scavenger receptors on the cell surface to foam cells without negative feedback [13].

The scavenger receptor SR-PSOX was firstly discovered by Shimaoka research group in 2000 [14]. Further studies show that the protein of SR-PSOX exists in either a membrane-bound or a soluble form. SR-PSOX in the membrane-bound form is a type I transmembrane glycoprotein with 254-amino acids, consisting of CXC chemokine, mucin stalk, transmembrane and cytoplasmic domains [15]. The soluble form of SR-PSOX, which serves as a chemokine CXCL16, is generated from its membrane form by the protease mediated cleavage [16-20]. The membrane form of SR-PSOX can bind and internalize oxLDL and phosphatidylserine, not native or acetylated LDL [14]. Similar to several other scavenger receptors, SR-PSOX is able to mediate adhesion and phagocytosis of Gram negative and positive bacteria by professional antigen-presenting cells [21,22]. Recently a study has showed that CXCL16^-/- ^LDLR^-/- ^mice develop larger atherosclerotic lesions, when compared with LDLR^-/- ^mice, which is expected to be atheroprotective [23]. More interestingly, Lehrke et al. has reported that patients with coronary artery disease (CAD) have a higher level of SR-PSOX [24] although Lieshout et al. indicates that the level of SR-PSOX in serum is not associated with the severity of CAD [25]. Obviously the relevance of the serum SR-PSOX and CAD needs to be further investigated.

SR-PSOX is the first scavenger receptor that has been identified as a chemokine [14]. The extracellular part of SR-PSOX consists of two distinct domains: the CXC chemokine domain and the mucin stalk domain. Shimaoka et al. have shown that only the chemokine domain of SR-PSOX is required for the recognition of bacteria and binding of oxLDL, however, bacterial phagocytosis via SR-PSOX is inhibited by oxLDL [21]. In addition, the replacement of some conserved basic amino acid residues in the chemokine domain of SR-PSOX with alanine significantly impaired the activities of bacterial phagocytosis, oxLDL uptake and the chemotaxis of CXCR6-expressing cells [26]. To examine whether the nonconservative basic amino acids in the chemokine domain are critical for the function of SR-PSOX, we generated a series of mutants of human SR-PSOX by replacing a single basic amino acid residue in the chemokine domain with alanine. Furthermore, we overexpressed these mutants in 293T cells and subsequently examined the uptake of oxLDL, phagocytosis of bacteria and adhesive activity. In this report, we demonstrate that the basic amino acids in the nonconservative region of the chemokine domain play a central role in the uptake of oxLDL, phagocytosis of bacteria and adhesive activity of SR-PSOX. Our findings provide new insight into the structure and function of SR-PSOX and the information may be useful for the development of drug target of atherosclerosis.

## Materials and methods

Plasmid pEGFP-N3 was purchased from Clontech Laboratories (Mountain View, CA, USA). Restriction enzymes EcoR I and BamH I, and T4 DNA ligase were obtained from Fermentas (USA). Plasmid DNA purification kits were purchased from Omega (USA). Trizol reagent and Alexa Fluor 594-labeled E.coli were purchased from Invitrogen (USA). DiI-oxLDL was obtained from Kalen Biomedical (USA). Goat anti-human SR-PSOX antibody and APC-labeled rat anti-human SR-PSOX antibody were obtained from R&D (USA).

### Cell culture

The human monocyte cell line THP-1 (Shanghai Institute of Cell Biology, Chinese Academy of Sciences, Shanghai, China) was grown in RPMI 1640 medium supplemented with 10% FBS, 100 μg/mL of streptomycin and 100 U/mL of penicillin. The human embryonic kidney cell line 293T (Shanghai Institute of Cell Biology, Chinese Academy of Sciences, Shanghai, China) was cultured in high glucose DMEM containing10% FBS, 100 μg/mL of streptomycin and 100 U/mL of penicillin. All the cells were maintained in a humidified 5% CO_2 _atmosphere at 37 °C. The medium was replaced every 2-3 days during the time of culture.

### Generation of SR-PSOX expression vectors

THP-1 cells were stimulated by 100nM PMA for 24 hours and the total RNA was extracted by Trizol reagent according to the instructions of the producer. Human SR-PSOX (GenBank accession number NM_022059) open frame was amplified by PCR from cDNA generated by reverse transcription of mRNA. The forward primers included a restriction site for EcoR I was 5'-CCG GAA TTC ATG TCT GGG AGT-3'. The reverse primer included a restriction site for BamH I was 5'-CGC GGA TCC GGT ATT AGA GTC-3'. The PCR product was cloned into the expression vector pEGFP-N3 (Invitrogen). The correct clone was confirmed by sequencing.

### Site-directed mutagenesis of SR-PSOX

The mutants of SR-PSOX were generated by the overlapping-extension PCR method. The technique was based on two rounds of PCR amplifications using two different flanking regions and two internal primers designed to generate the desired mutations. During the first PCR round, the internal primers and flanking primers were used to generate double stranded portions of the target DNA sequence that contains the desired mutations. During the second PCR round, those double-stranded DNA fragments and flanking primers were utilized to generate a mutated product corresponding to the total length of the target sequence. All mutations of SR-PSOX in the study used were performed with the above approach. All the primers needed were designed by Primer Premier 5.0 software. The specific primer pairs used for the mutagenesis were as follows (small letters represent mutational nucleotides): R62A, 5'-CGG AAG AAA TTg cTT TAC CAC AAT-3' and 5'-ATT GTG GTA AAg cAA TTT CTT CCG-3'; R78A, 5'-CTC AGG TGT TTa gcG AGA CGA TTC AT-3' and 5'- ATG AAT CGT CTC gct AAA CAC CTG AG-3'; H80A, 5'- GTA AGC TCT CAG Agc TTT CCG GAG AC -3' and 5'- GTC TCC GGA AAg cTC TGA GAG CTT AC -3'; R82A, 5'- GAT GGT AAG CTg cCA GGT GTT TCC -3' and 5'- GGA AAC ACC TGg cAG CTT ACC ATC -3'; H85A, 5'- GTA TAG ACA CCG Agc GTA AGC TCT CAG -3' and 5'- CTG AGA GCT TAC gcT CGG TGT CTA TAC -3'; K105A, 5'- ACC CAT GGG TCa gcG TTG CCC CCA C -3' and 5'- GTG GGG GCA ACg ctG ACC CAT GGG T -3'; K119A, 5'- GTC CAC ATT CTg cGA GAT CAA GAC -3' and 5'- GTC TTG ATC TCg cAG AAT GTG GAC -3'; H123A, 5'- CCG AGT AAG CAg cTC CAC ATT CTT -3' and 5'- AAG AAT GTG Gag cTG CTT ACT CGG -3'. PCRs were performed using *pfu*DNA polymerase (Tiangen Biotech, China) according to the manufacturer's protocol. The mutants of SR-PSOX were cloned into the expression vector pEGFP-N3. The correct mutations of SR-PSOX were confirmed by sequencing.

### Transfection of SR-PSOX recombinant plasmids

293T cells were seeded on coverslips and cultured in 24-well plates in DMEM medium overnight to allow cells to grow to 60-70% confluence at the time of transfection. For the transfection, U-fectin Transfection Kit (Plurigen, China) was used according to the manufacturer's instructions. Briefly, 0.5 μg DNA of SR-PSOX recombinant plasmid was diluted in 50 μl RPM1640 medium. 1.5 μl U-fectin was diluted in 25 μl U-fectin buffer mixed with 50 μl diluted DNA solution, and incubated for 20 minutes at room temperature. The mixture was then added to the well with 0.5 ml of fresh DMEM medium. The transfected cells were cultured for 24 hours before carrying further experiments.

### Analysis of SR-PSOX protein expression by flow cytometry

Transfected cells were harvest into FACS tubes by using 0.5 mM EDTA, and washed twice with FACS buffer (1 × PBS containing 5% FBS and 0.01%NaN_3_). Cells were incubated with APC-labeled rat anti-human SR-PSOX antibody for 40 minutes at 4°C after cells were blocked with mouse serum, followed by washing twice with FACS buffer. Stained cells were assayed by Flow Cytometry (BD Calibur, USA) after adding FACS fixed buffer (FACS buffer containing 1% paraformaldehyde).

### Analysis of SR-PSOX protein expression by fluorescence microscopy

Cells were cultured on coverslips after 24 hours transfection and then fixed with 4% paraformaldehyde for 20 minutes at room temperature. After fixation, cells were blocked with PBST (containing 1% bovine serum albumin and 0.3M glycine) for 30 minutes at room temperature. Cells were then washed with PBS and incubated with the goat anti-human SR-PSOX antibody (diluted in 1% bovine serum albumin/PBST) overnight at 4°C, followed by washing with PBST. Cells were then incubated with Cy3-labeled donkey anti-goat secondary antibody (Beyotime, China) for 1 hour at room temperature in dark. After washing, the cells on coverslides were mounted in glycerol and sealed with nail polish. Evaluation of SR-PSOX expression on the surface of cell was performed by Confocal Microscope (Leica TCS SP5X Microsystems, Germany).

### Uptake of oxLDL

Transfected cells were assayed for oxLDL uptaking. The culture medium was replaced with 250 μl fresh culture medium DMEM in each well and cells were incubated with 20 μg/ml Dil-oxLDL for 4 hours at 37°C. Cells were then washed twice with PBS and collected into FACS tubes with 0.25% trypsin. The cells were assayed by Flow Cytometry after washing twice with FACS buffer.

### Phagocytosis of bacteria

Transfected cells were incubated with Alexa Fluor 594-labeled E.coli (3 × 10^6 ^cells/ml) for 4 hours at 37°C. Cells were then washed with FACS buffer and detached with trypsin. The cells were collected into FACS tubes and washed twice with FACS buffer again. The number of cells internalized with Alexa Fluor 594-labeled bacteria was quantified by Flow Cytometry.

### Assays of cell adhesion

Transfected cells were washed with PBS before incubating with 0.5M EDTA for 10 minutes at 37°C. Cells were then washed again with PBS and fixed with 4% paraformaldehyde for 15 minutes. Being washed with distilled water, cells were stained with 0.1% crystal violet staining solution for 20 minutes at room temperature. The cells were washed again and allowed to air dry before cells were incubated with 10% acetic acid for 20 minutes with shaking. The OD values for crystal violet staining were measured at 595 nm by Microplate Reader (ELX 800 Bio Tek, USA).

### Statistical analysis

All experiments were repeated at least three times respectively. The data shown in all figures are expressed as the mean ± SEM of values from independent experiments. The paired *Student-t *test was used to evaluate the statistical significance of differences between experimental groups. Samples with P < 0.05 were considered significantly different.

## Results

### Expression of wild-type SR-PSOX and mutants in 293T Cells

**Figure 1:**
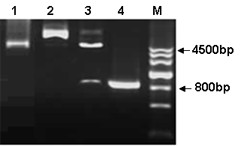
**Construction of SR-PSOX fragment into pEGFP-N3 expression vector**. Constructed products were analysed by 1% agarose gel to confirm the right size of fragment. Lane 1, empty pEGFP-N3 vector only (4700bp). Lane 2, wild type of SR-PSOX fragment was inserted in to the vector as pEGFP-N3-SR-PSOX construct (5541bp). Lane 3, pEGFP-N3-SR-PSOX construct was digested with EcoR I and BamH I to form the right size of two bands (4700bp and 841bp). Lane 4, the right size of full-length of SR-PSOX fragment was amplified after PCR reaction. M, DNA ladder.

**Figure 2:**
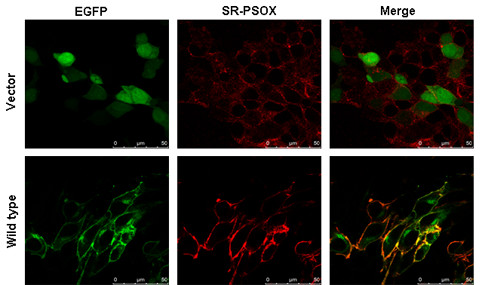
**The transfected 293T cells were expressing pEGFP and SR-PSOX proteins detected by confocal microscopy**. 293T cells were transiently transfected with pEGFP-N3 empty vector or pEGFP-N3-SR-PSOX construct (wild-type SR-PSOX cloned into pEGFP-N3 vector) for 24 hours respectively. The fluorescence images of the cells were observed under confocal microscope after the cells were immunostained with APC-labeled rat anti-human SR-PSOX antibody. Top panel (vector), the cells were transfected with pEGFP-N3 empty vector alone. Bottom panel (wild type), the cells were transfected with pEGFP-N3-SR-PSOX construct.

The scavenger receptor SR-PSOX is a 30 kDa type I transmembrane glycoprotein. The coding sequence of SR-PSOX (837 bp) was subcloned into pEGFP-N3 vector (Figure 1). All the SR-PSOX mutants were constructed as described in materials and methods, and confirmed by sequencing. To verify if recombinant plasmid pEGFP-N3-SR-PSOX could be normally expressed on the surface of cells, a pEGFP-N3 empty vector and pEGFP-N3-SR-PSOX were transfected into 293T cells respectively. After 24 hours transfection, the cells were stained with anti-SR-PSOX antibody, counter stained with a Cy3-conjugated secondary antibody, and the images were then directly viewed under a fluorescent microscope. As shown in Figure 2, EGFP was well expressed in the cells transfected with either of the recombinant plasmids. As expected, SR-PSOX was expressed on the plasma membranes.

**Figure 3:**
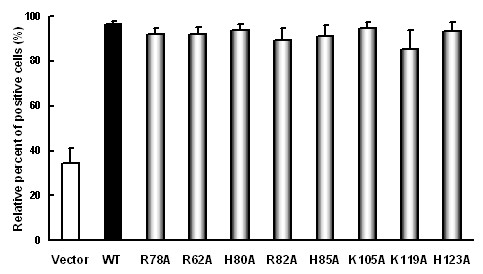
**Expression levels of SR-PSOX on the surface of transfected 293T cells**. 293T cells were transiently transfected with pEGFP-N3 empty vector, wild-type SR-PSOX and different mutant constructs respectively. After 24 hours transfection, the cells were harvested and stained with APC-labeled anti-SR-PSOX antibody. The expression levels of SR-POSX on the surface of the cells in each group were measured by FACS. The data are represented as the mean ± SEM by the percentage of SR-PSOX-positive cells in the EGFP-positive cells from three independent experiments.

Shimaoka et al. first showed that conserved basic amino acids in the chemokine domain of SR-PSOX are critical for the cells migration, phagocytosis and ox-LDL uptake [26]. To examine whether the non-conservative basic amino acids in the chemokine domain of SR-PSOX are the recognition sites of SR-PSOX for bacteria, oxLDL and adhesive activity, several basic amino acids of human SR-PSOX in the chemokine domain were selected with the help of bioinformatics for mutagenesis. These basic amino acid residues, including arginine 62, histidine 80, arginine 82, histidine 85, lysine 105, lysine 119 and histidine 123, located in the non-conservative region of the chemokine domain in human SR-PSOX were replaced with alanine (designated as R62A, H80A, R82A, H85A, K105A, K119A and H123A, respectively). Arginine 78 (R78A) was set as a control (reported as R59A in Ref. 26). SR-PSOX wide-type and mutant constructs were transiently transfected into 293T cells and the expressing level of the proteins in the cells was determined by FACS analysis (Additional file 1: Figure S1). Obviously the SR-PSOX proteins were evenly expressed on the surface of the cells as determined by flow cytometry analysis after immunostaining (Figure 3).

### Replacements of basic amino acid residues in SR-PSOX alter DiI-oxLDL uptake in the transfected 293T Cells

**Figure 4:**
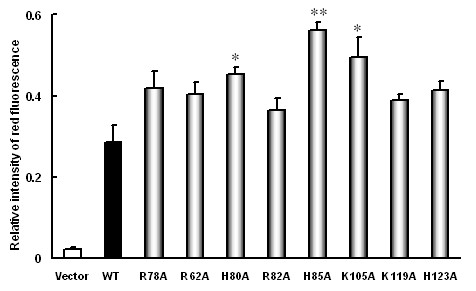
**Uptake of DiI-oxLDL by the transfected 293T cells**. 293T cells were transiently transfected with pEGFP-N3 empty vector, wild-type SR-PSOX and different mutant constructs respectively. After 24 hours transfection, cells were incubated with DiI-oxLDL for 4 hours at 37°C. The relative intensity of red fluorescence represents the ability of DiI-oxLDL uptaked by the cells. The results show the mean ± SEM of the intensity of red fluorescence in the EGFP-positive 293T cells from three independent experiments. *: P < 0.05, **: P < 0.01, each compared to the wild-type SR-PSOX (WT).

Since oxLDL is a ligand for SR-PSOX, replacement of certain amino acid in the non-conservative region of the chemokine domain of SR-PSOX may result in altering its capability to oxLDL uptake. To test the hypothesis, 293T cells were transfected with SR-PSOX wide-type and mutant plasmids. After transfection for 24 hours, the cells were incubated with Dil-oxLDL and the uptake of DiI-oxLDL in the cells was analysed by FACS (Additional file 2: Figure S2). As shown in Figure 4, the uptake of DiI-oxLDL by H80A, H85A and K105A mutant-expressed cells was increased by 58%, 96% and 72% respectively compared with that of wild-type SR-PSOX, and the enhancements were statistically significant. However, the cells expressing other mutants only slightly increased the uptake of DiI-oxLDL, suggesting that mutation of H80, H85 and K105 is very important for the cell to absorb oxLDL. These results implicate that mutagenesis of the basic amino acid in the non-conservative region of the chemokine domain may contribute to the atherogenic process.

### Activities of bacterial phagocytosis could be changed by replacements of basic amino acid residues in SR-PSOX

**Figure 5:**
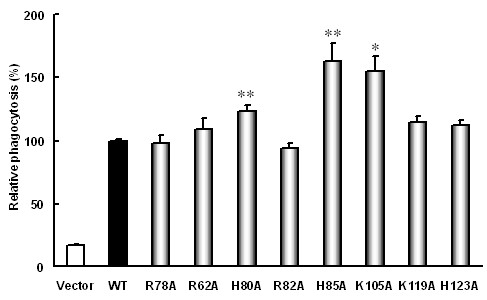
**Phagocytosis of bacteria by the transfected 293T cells**. 293T cells were transiently transfected with pEGFP-N3 empty vector, wild-type SR-PSOX and different mutant constructs respectively. After 24 hours transfection, the cells were incubated with Alexa Fluor 594-labeled *E. coli *for 4 hours at 37°C. The mean values of phagoctosed bacterial are represented as the intensity of Alexa Fluor 594 fluorescence in the EGFP-positive 293T cells. The values are relative to the wild-type SR-PSOX group set as 100%. The data show the mean ± SEM from four independent experiments. *: P < 0.05, **: P < 0.01, each compared to wild-type SR-PSOX (WT).

SR-PSOX plays an important role in facilitating uptake of various pathogens by APCs through its chemokine domain [27]. To determine whether mutation of any basic amino acid in the non-conservative region of the chemokine domain impacts phagocytosis of the cells, 293T cells transfected with wild-type and mutant types of SR-PSOX were incubated with Alexa Fluor 594-labeled *E. coli*. and internalized bacteria in the cells were quantified by FACS (Additional file 3: Figure S3). Interestingly the phagocytotic activity of the cells expressing H80A, H85A and K105A mutant was remarkably increased when compared with the cells expressing wild-type SR-PSOX (Figure 5). Taken together, our results suggest that H80, H85 and K105 may be necessary for SR-PSOX to function as a gate guard to prevent foreign molecules from getting into a cell.

### Replacements of basic amino acid residues in SR-PSOX could reduce the adhesive activity in the transfected 293T cells

**Figure 6:**
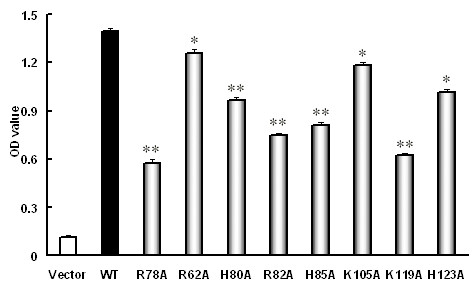
**Cells adhesion ability in the transfected 293T cells**. 293T cells were transiently transfected with pEGFP-N3 empty vector, wild-type SR-PSOX and different mutant constructs respectively. After 24 hours transfection, the cells were incubated with EDTA for 10 minutes at 37°C. The number of adhesion cell was quantified by crystal violet stain. OD value was used to measure the adhesive ability on the cells. The data show the mean ± SEM from three independent experiments. *: P < 0.05, **: P < 0.01, each compared to wild-type SR-PSOX (WT).

To examine whether these basic amino acids are important in the non-conservative region of the SR-PSOX chemokine domain for the adhesive activity of cells, 293T cells expressing wild type and mutants of SR-PSOX were incubated in the presence or absence of EDTA for 10 minutes at 37 °C, and the adhesion cells were quantified by crystal violet stain. To our surprise, the cells expressing all the SR-PSOX mutants (R78A, R62A, H80A, R82A, H85A, K105A, K119A and H123A) showed significant reduction of adhesive activities when compared with the cells expressing wild-type SR-PSOX. As shown in Figure 6, the highest reduction of adhesive activity among these mutants was about 58.5%. Thus, the results suggest that all of the basic amino acid residues in the non-conservative region of the SR-PSOX chemokine domain may be critical for cell adhesion.

## Discussion

SR-PSOX functions as a membrane receptor for multiple ligands including oxLDL, phosphatidylserine and bacteria. On the other hand, the soluble form of SR-PSOX, produced by proteolytic cleavage of the membrane-bound form, is an interferon-gamma-regulated chemokine CXCL16 [14,21,22]. Structurally, SR-PSOX consists of CXC chemokine, mucin stalk, transmembrane and cytoplasmic domains [15]. Shimaoka et al. have shown that the chemokine domain of SR-PSOX is uniquely required for the recognition of bacteria and is responsible for phagocytosis of macrophages. Interestingly, it has been found that both bacteria and oxLDL compete for binding of SR-PSOX [21]. In this study, we have found that the basic amino acid residues at non-conservative region in the chemokine domain are very important for the function of SR-PSOX. Mutation of these individual amino acids, in particular H80, H85 and K105, resulted in significantly increasing the activities of oxLDL uptake and bacterial phagocytosis. Interestingly, mutagenesis analysis revealed that all of these amino acids selected from in the non-conservative region of the SR-PSOX chemokine domain are necessary for cell adhesion. Clearly, our results provide a novel spectrum about the structure and function of SR-PSOX.

CX3CL1 is another transmembrane chemokine with similar functions as SR-PSOX. It has been reported that both the chemotaxis and adhesion functions of the cells overexpressing CX3CL1 are critically impaired by the replacement of the basic amino acid residues in the chemokine domain of CX3CL1 with alanine [27]. Similarly, Shimaoka et al. have also found that conversion of any basic amino acid residue, in particular R59, R67 and R73, with alanine in the conserve region of the chemokine domain of SR-PSOX significantly reduced activities of bacterial phagocytosis, oxLDL uptake and the chemotaxis of the cells expressing the mutants when compared with wild-type SR-PSOX [26], suggesting that the individual basic amino acid residue in the conserve region of the chemokine domain of SR-PSOX is also important for the recognition of oxLDL, bacteria and adhesion. However, Shimaoka's group has reported that the mutant R78A did not significantly change the activities in oxLDL uptake and bacterial phagocytosis, which is controversial with our observation [26]. There are two possibilities that may cause the difference: (1) FACS was used in our study to detect the uptake of oxLDL while Shimaoka's group used fluorescence microscopy. The FACS analysis is considered to be more sensitive than microscopy analysis; (2) we incubated Alexa Fluor 594-labeled *E. coli *for 4 hours at 37 °C while Shimaoka's group only incubated FITC-labeled *E. coli *for 1 hour at 37 °C. In our system, we hardly detected the phagocytosis of *E. coli *after incubation for 1 hour at 37 °C. These indicated that phagocytosis effect is time dependent.

The extracellular part of SR-POSX is composed of chemokine domain and mucin domain. It has been reported that CX3CR1 also has the two distinct domains that critically influence cells adhesion [28]. Our results are consistent with the observation. Taken together, it is obvious that a highly non-overlapping set of basic amino acid residues in the non-conservative region of the chemokine domain are important for cell adhesion.

## Conclusions

The cell model for studying the functions of SR-PSOX has been successfully established in this study. The results reveal that non-conservative basic amino acids in the chemokine domain are critical for the functions of SR-PSOX. Further studies will be needed to investigate the occurrence of mutagenesis in these basic amino acids in the clinical specimens, which may provide direct evidence that mutation of a specific basic amino acid in the non-conservative region of the chemokine domain of SR-PSOX contributes to the development of AS. The information obtained from this study may be useful for the design of therapeutic agents that target SR-PSOX for the treatment of AS.

## Competing interests

The authors declare that they have no competing interests.

## Authors' contributions

YD and LY conceived the idea and designed the study. WL performed experiments. LY participated in the experiments related to flow cytometer. LY and CC performed the statistical analysis and interpretation of the data. YD and WL drafted the manuscript. All authors read and approved the final manuscript.

## Supplementary Material

Additional File 1 **Figure S1: Expression of SR-PSOX receptor on the surface of transfected 293T cells**. 293T cells were transiently transfected with empty vector, wild-type SR-PSOX and different mutant constructs respectively. After 24 hours transfection, the cells were harvested and assayed. The cells were stained with APC-labeled anti-SR-PSOX antibody, and SR-PSOX positive cells were then detected by FACS. The data shown represent one typical experiment from three independent experiments. Click here for file

Additional File 2 **Figure S2: Uptake of DiI-oxLDL by transfected 293T cells**. 293T cells were transiently transfected with empty vector, wild-type SR-PSOX and different mutant constructs respectively. After 24 hours transfection, the cells were incubated with DiI-oxLDL for 4 hours at 37 °C, and then DiI-oxLDL positive cells were measured by FACS. The data shown represent one typical experiment from three independent experiments Click here for file

Additional File 3 **Figure S3: Phagocytosis of bacteria by transfected 293T cells**. 293T cells were transiently transfected with empty vector, wild-type SR-PSOX and different mutant constructs respectively. After 24 hours transfection, the cells were incubated with Alexa Fluor 594-labeled *E. coli *for 4 hours at 37°C, and the *E. coli *positive cells were counted by FACS. The data shown represent one typical experiment from four independent experiments. Click here for file
