# Supplementary figures and images for: Function modification of SR-PSOX by point mutations of basic amino acids
Source: Lipids Health Dis. 2011 Apr 15;10:59. doi: 10.1186/1476-511X-10-59 (PMC3102630; doi:10.1186/1476-511X-10-59)

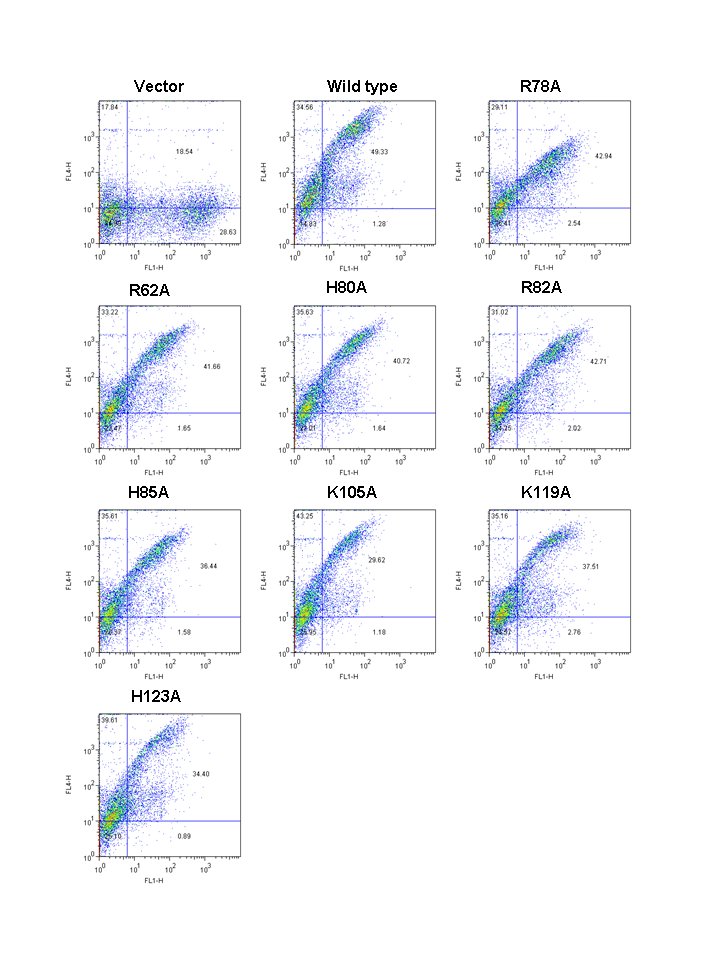

Supplement: Additional File 1 — Figure S1: Expression of SR-PSOX receptor on the surface of transfected 293T cells. 293T cells were transiently transfected with empty vector, wild-type SR-PSOX and different mutant constructs respectively. After 24 hours transfection, the cells were harvested and assayed. The cells were stained with APC-labeled anti-SR-PSOX antibody, and SR-PSOX positive cells were then detected by FACS. The data shown represent one typical experiment from three independent experiments. [file 1476-511X-10-59-S1.TIFF]

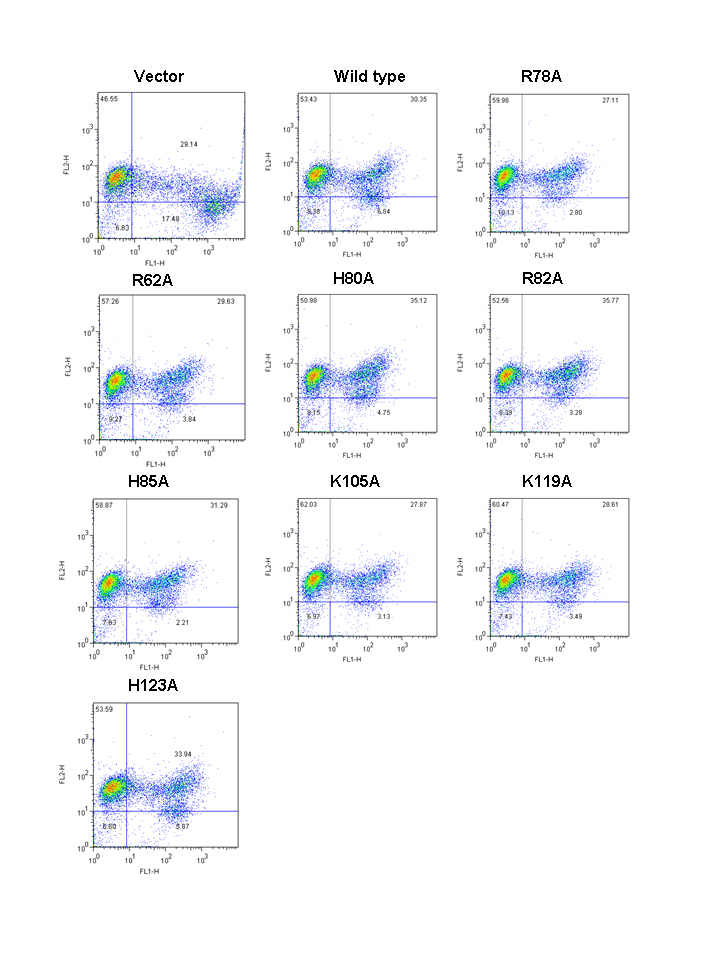

Supplement: Additional File 2 — Figure S2: Uptake of DiI-oxLDL by transfected 293T cells. 293T cells were transiently transfected with empty vector, wild-type SR-PSOX and different mutant constructs respectively. After 24 hours transfection, the cells were incubated with DiI-oxLDL for 4 hours at 37 °C, and then DiI-oxLDL positive cells were measured by FACS. The data shown represent one typical experiment from three independent experiments [file 1476-511X-10-59-S2.TIFF]

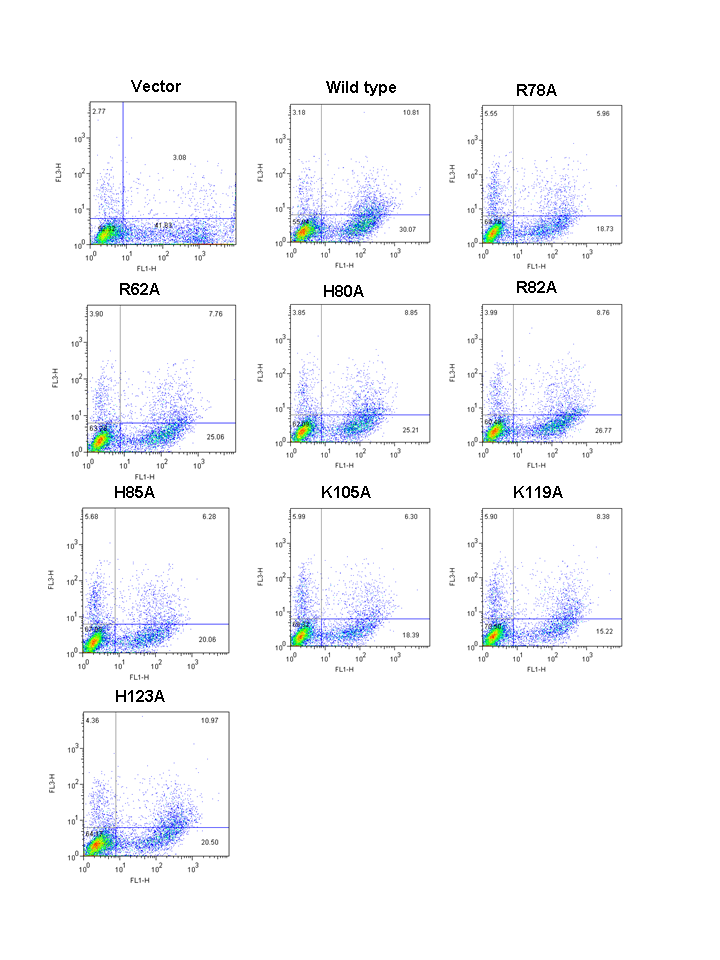

Supplement: Additional File 3 — Figure S3: Phagocytosis of bacteria by transfected 293T cells. 293T cells were transiently transfected with empty vector, wild-type SR-PSOX and different mutant constructs respectively. After 24 hours transfection, the cells were incubated with Alexa Fluor 594-labeled E. coli for 4 hours at 37°C, and the E. coli positive cells were counted by FACS. The data shown represent one typical experiment from four independent experiments. [file 1476-511X-10-59-S3.TIFF]
